# Telemedicine in Endourology for Patient Management and Healthcare Delivery: Current Status and Future Perspectives

**DOI:** 10.1007/s11934-024-01224-6

**Published:** 2024-07-09

**Authors:** Ali Talyshinskii, Nithesh Naik, B. M. Zeeshan Hameed, Gafour Khairley, Princy Randhawa, Bhaskar Kumar Somani

**Affiliations:** 1 Department of Urology and Andrology, Astana Medical University, Astana, Kazakhstan; 2 Department of Mechanical and Industrial Engineering, Manipal Institute of Technology, Manipal Academy of Higher Education, Manipal, Karnataka India; 3 Department of Urology, Father Muller Medical College, Mangalore, Karnataka India; 4 Department of Mechatronics Engineering, Manipal University Jaipur, Jaipur, Rajasthan India; 5 Department of Urology, University Hospital Southampton NHS Trust, Southampton, UK

**Keywords:** Telemedicine, Telehealth, Endourology, Urolithiasis, BPH

## Abstract

**Purpose of Review:**

Researchers have examined how telemedicine affects endourological patients. This review analyzes the literature to determine telemedicine's benefits and limitations in endourology.

**Recent Findings:**

Many studies were devoted to describing the effect of telemedicine on endourological patient satisfaction, optimization of the clinical decision-making among patients with kidney and ureteric stones, the effectiveness of telemedicine in the management of patients with indications for PCNL, follow-up for patients with urolithiasis and describing financial effectiveness for the patients after BOO surgery. The authors describe phone calls, video calls, and online booking platforms as used as telemedicine technology. However, several concerns also exist, such as the necessity of internet connections and appropriate devices, different receptivity among certain subgroups, data safety, and different regulatory environments among countries.

**Summary:**

Telemedicine offers the potential to reduce patient travel time, expedite decision-making, and save costs in endourology. However, its everyday implementation is challenging due to various obstacles faced by patients and providers, hindering the realization of its full potential and necessitating a systematic approach to problem-solving.

## Introduction

**Fig. 1:**
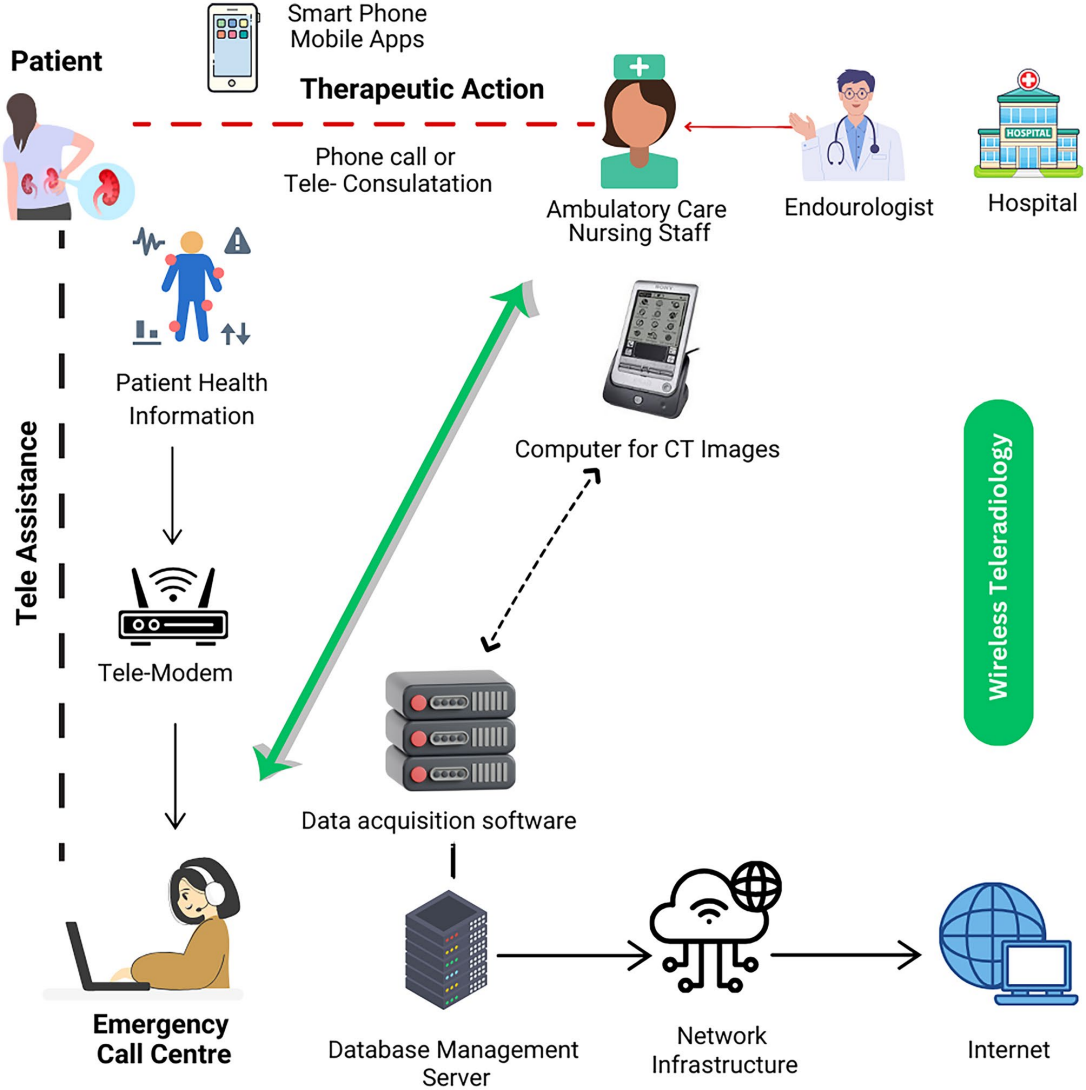
Schematic architecture of telemedicine implementation for endourological patients

A rapid increase in the use of digital healthcare technologies across all medical specialties is one of the defining characteristics of the modern era [1]. There are a few different domains that are encompassed by this phrase, the most well-known and commonly used being telemedicine [2]. This type of technology typically does not require specific devices and software, something that explains the ease of use in terms of the idea. Despite this, telemedicine was not as thoroughly researched in the field of urology as it was in other medical specialties. It was also thought to play a limited role up until the pandemic of coronavirus disease-19 (COVID-19) in the year 2020. During this time, the urological community witnessed all of the advantages that telemedicine offers for both general practitioners and specialists [3]. Despite the fact that it is possible to restore workflow to the way it was before the pandemic, this technology is still a promising and useful instrument in the field of urology. Figure 1, shows the schematic architecture of telemedicine implementation for endourological patients.

The usefulness of telemedicine technology has been established in the fields of pediatric urology, urogynecology, and uro-oncology [4–6]. The advantages and disadvantages of telemedicine should be evaluated for each patient cohort on an individual basis, given the fact that medicine is a field that is highly individualized and focused on the patient. Several studies have been conducted to investigate the impact that telemedicine has on endourological patients [7]. Furthermore, its notion in endourology has been recognized since the late twentieth century, when Hayes et al. published the findings of a prototype teleconsultation study that lasted for six months and linked Georgetown University Medical Center (GUMC) in Washington, District of Columbia, with City Hospital in Martinsburg, West Virginia [8]. Therefore, the objective of this research is to conduct a comprehensive literature analysis to ascertain the advantages and limitations that telemedicine offers and the challenges faced in the field of endourology.

## Material and Methods

Search: In February 2024, the systematic publication search was done in several databases, including ACM Digital Library, CINAHL, IEEE Xplore, PubMed, and Google Scholar via Boolean operators with the use of the following terms: “telemedicine”,”telehealth”, “virtual clinic”, “phone call”, “video call”, “endourology”, “urolithiasis”, “kidney calculi”, “kidney stone disease”, “KSD”, “benign prostate hyperplasia”, “BPH”, “bladder outlet obstruction", “BOO”, “consultation” and “follow-up”. To be more specific in endourological topic, the search was focused on urolithiasis and bladder outlet obstruction (BOO) due to benign prostate hyperplasia (BPH), as being more often non-oncological indications for the endourological treatment.

### Inclusion Criteria

Description of the usage of telemedicine in the diagnosis, treatment, and follow-up of endourological patients; the presence of the description of telehealth methodology used, number of patients involved, results in the percentage (%) or count and English-written papers. Considering the potential low number of studies the search was done both without publication date restriction and regardless of the study design.

### Exclusion Criteria

Papers not in the English language, non-accessibility of full papers, reviews, and studies describing telemedicine usage in urology without subgroup analysis focusing on endourological patients.

### Studies Process

Two reviewers (A.T. and N.N.) independently identified all papers. All studies fitting the inclusion criteria were selected for full review. If there was disagreement or discrepancy, the senior author (B.K.S.) made the final decision.

### Data Extraction and Analysis

We reviewed studies and extracted information related to the objective, type of telemedicine approach used, endourological nosology (urolithiasis, bladder outlet obstruction, or both), number of endourological patients involved, results in the percentage or direct count, outcomes, and validation type. This study was reported according to the Preferred Reporting Items for Systematic Reviews and Meta-Analysis (PRISMA) guidelines. We followed the PRISMA Checklist. As is a narrative literature review, the registration in dedicated sites such as PROSPERO was not deemed necessary.

## Results

**Fig. 2:**
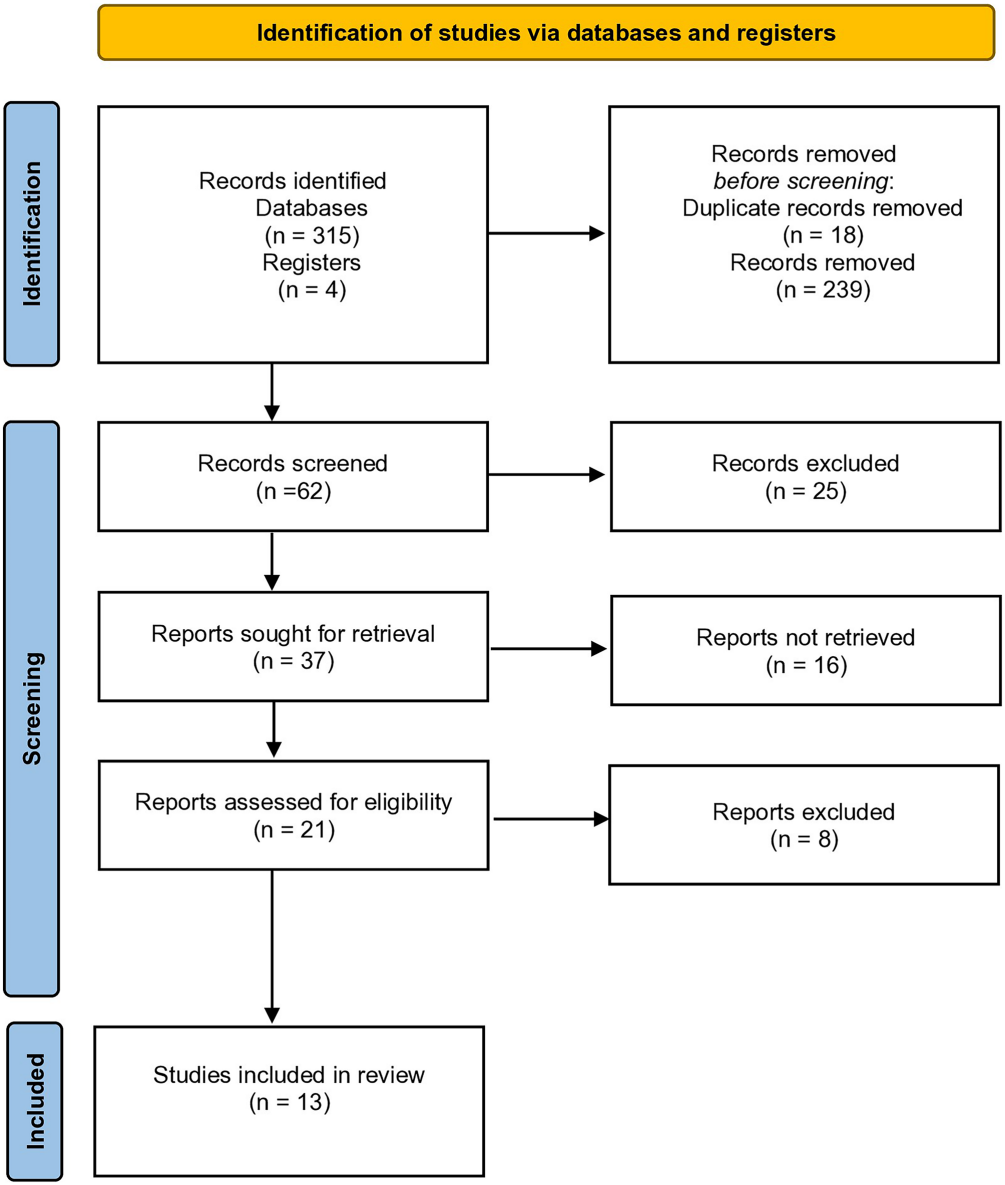
PRISMA flowchart exploring the literature of telemedicine in endourology

As a result of the literature search in accordance with the inclusion criteria, from 319 articles, 13 publications were included in the final analysis (Fig. 2), of which two [9, 10], three [11–13], five [14–18], and one each [19–21], were devoted to determining the number of endourological patients who can be managed by telemedicine, endourological patient satisfaction, optimization the clinical decision making among patients with kidney and ureteric stones, the effectiveness of telemedicine in the management of patients with indications for PCNL, follow-up for patients with urolithiasis and describing financial effectiveness for the patients after BOO surgery, respectively. Median number of cases was 80 (range: 11–1008). Notably, the lowest patient number was used in the earliest study, done in 2005 [16]. Phone calls [9, 13, 14, 16–18, 20, 21], video calls [11, 19], both [10, 12], and online booking platform [15] were used in eight, two, two, and one study respectively. Urolithiasis and BPH cases were separately considered by 10 [10, 12–20] and one [21] study, whereas endourological nosology was not mentioned in two papers [9, 11]. Merits of telemedicine were indicated by all studies, whereas only six of 13 provided its disadvantages [10, 11, 13, 16–18] (Table 1).

**Table 1:** Details of the included studies, results and pros and cons of studies relating to telemedicine in endourology

Authors	year	purpose	Type of telehealth	Endourological nosology	Number of endourology patients	results	pros	cons
Turcotte et al. [9]	2020	To reveal the percentage of urological cases that can be managed by telemedicine	Phone call	Not specified	152	86.8% were treated completely	-Telemedicine is a viable option for initial or follow-up visits -Particularly useful for patients with geographical or socioeconomic barriers. -Endourological patients are more susceptible to telemedicine.	-none
Somani et al. [10]	2020	To evaluate the impact of a 7-week lockout on the operations of all planned outpatient clinics and urgent treatments.	Phone call / Video call	Urolithiasis	193	94 (48.7%) of 193 patients with stones were transferred to telemedicine.	- Urologists and hospitals can both benefit from prioritizing patients who require emergent care.	-Telemedicine requires the instruction of clinicians and other healthcare professionals prior to its pervasive adoption and utilization.
Glassman et al. [11]	2018	To assess if some urological diagnoses are better suited for telemedicine management.	Video call	Not specified	41	The average satisfaction rating among doctors and patients was 4.9 and 4.2 out of 5, respectively.	Video consultations can be employed for a diverse range of medical conditions, yielding high levels of patient contentment, irrespective of the geographical proximity to a healthcare center.	- Complicated use with increased age - Dedicated equipment is required
Posid et al. [12]	2022	To assess the level of satisfaction among endourological patients participating in a telehealth program.	Phone call / Video call	Urolithiasis	52	The patients expressed high levels of satisfaction with their telehealth consultation, with an average score of 6.3 out of 7.	Patients who had telemedicine consultations for kidney stones and pain expressed high levels of satisfaction with their telemedicine appointments, despite the option of scheduling in-person sessions.	-n/a
Heeno et al. (13)	2021	to collect post-COVID-19 urology patients' feedback on their telemedicine experience during lockdown	Phone call	Urolithiasis	122	85.2% of patients were satisfied with telemedicine only 36.4% would prefer telephone consultations in the future	- high patient satisfaction during lockdown	-different response between age groups - lack of personal contact is patients’ primary reason for a negative attitude to telephone consultations -Dedicated equipment is required -Technical skills are necessary
Ong et al. [14]	2020	To determine if ureteric colic patients' telemedicine service reduces unnecessary face-to-face visits.	Phone call	Urolithiasis	1006	Stable monthly clinic consultation savings ranged from 52.9% to 89.5%.	-Reduce face-to-face consultations and review time for ureteric colic patients without compromising safety. - Increase space availability in clinics and parking areas - Decrease the emission of greenhouse gases	-none
Cullen et al. (15)	2023	To define the benefits of a virtual colic clinic for efficient care and reduced wait times for renal colic patients.	Online booking platform	Urolithiasis	55	Increased percentage from 25% to 82% of patients reviewed within 4 weeks.	-Improve ureteric stone management time for patients following BAUS recommendations using expectant management. -Reduce clinic review and stone treatment wait times.	-none
Johnston et al. [16]	2005	To assess the feasibility and diagnostic accuracy of wirelessly transmitting digital images to a handheld computer in acute renal colic patients.	Phone call + hand-held computer	Urolithiasis	11	Interpretation properly recognized 80% stone presence, 100% hydronephrosis, and 80% perinephric stranding.	Wireless teleradiology may help remote patients get acute renal colic evaluations quickly.	Telemedicine relies on the proficiency of a highly skilled physician to assess the patient.
Connor et al. (17)	2019	To assess a specialist-led acute ureteric colic virtual clinic (VC) pathway's clinical, budgetary, and environmental effects.	Phone call	Urolithiasis	1008	Saved £145,152 for Clinical Commissioning Groups. 15,085 patient trip kilometers were avoided.	-cut treatment decision time. - Increases clinic capacity - Lowers traditional clinics' cost load -Decreases carbon footprint.	This treatment is not suitable for all patients, especially those with significant comorbidities, complex pathology, language hurdles, or specific circumstances such as pregnancy.
Hughes et al. (18)	2021	To describe a nurse-led telephone-based virtual stone clinic (VSC) follow-up for asymptomatic renal calculi or high-risk kidney stone patients.	Phone call	Urolithiasis	290	132 patients (45.6%) remained in VSC. 93% clinic appointment cost decrease.	-Provides a safe follow-up -Allows reducing the cost of treatment	-Specific training of stuff is necessary
Aydogdu et al. (19)	2019	To examine the impact of telerounding on post-operative outcomes, patient satisfaction, and surgeon satisfaction in percutaneous nephrolithotomy patients.	Video call	Urolithiasis	80	VAS score for surgeon satisfaction in telerounding was 91±11.2, with 72.5% of patients expressing good satisfaction.	Adding telerounding to endourological patient care increases satisfaction for both patients and surgeons.	-none
Nevo et al. (20)	2020	To compare dietary 24-h urine parameters after office-based consulting (OC) with phone correspondence (PC).	Phone call	Urolithiasis	43	Improved urine volume after PC. Patients with both OC and PC had longer follow-up and more consults than those with only OC visits.	-Improved compliance with subsequent appointments. -Reduced waiting time. -Decreased expenses associated with visits. -Enhanced the adaptability and accessibility of consultations.	-none
Sarmah et al. (21)		Using IPSS measurement, create a nurse-led follow-up virtual clinic (VC) for BOO surgery patients.	Phone call	BPH	50	Total cost savings with VC amounted to £10,634	Telephone follow-up for BOO surgery based on IPSS is clinically safe and cost-effective	- none

Turcotte et al. surveyed all urologists practicing in the Quebec City region to get their views on the proportion of outpatient urological cases that might be fully handled using telemedicine outside of the COVID-19 pandemic [9]. Out of a total of 1679 appointments covering various urological fields, 152 were specifically associated with endourology, and the percentages for the different types of cases were 86.8% for complete, 9.9% for suboptimal, and 3.3% for incomplete management. Somani et al. examine the impact of a 7-week lockdown on all planned outpatient clinics and urgent operations [10]. Whenever feasible, all in-person appointments were converted to virtual telephone or video clinic appointments, except for patients who needed flexible cystoscopy and shockwave lithotripsy (SWL) treatments, who still attended in person. Out of a total of 193 patients with stone issues, 94 individuals (48.7%) were shifted to a telemedicine format.

Along with determining the number of endourology patients who can be safely assessed using telemedicine tools, it is important to ask them about their experience and identify factors that prevent telemedicine from being used in clinical practice. So, Glassman et al. conducted a study to evaluate patient satisfaction and determine if certain urological diagnoses are better suited for remote encounters [11]. The secondary aim was to assess patient satisfaction based on age and proximity to the clinic. The data indicates that the average satisfaction level among physicians was 4.9 (out of 5), while among patients it was 4.2. Similarly, Posid et al. conducted a sub-analysis of urologic telehealth patient satisfaction and kidney stone patients. 34.4% of patients utilized phone/audio, 45.0% used EPIC/MyChart video, and the rest used Doximity, UpDox, or other type of device [12]. Kidney stone patients (55.8%) were more likely to employ EPIC/MyChart video than other methods, and/or benign and oncology patients (p = 0.013). Overall, patients were very satisfied with their telemedicine consultation (M = 6.3/7, p < 0.001 vs. ‘neutral’), with kidney stone patients being somewhat more satisfied (p = 0.084). Heeno et al. evaluated patients' perception of their telephone consultation and their overall attitude toward the future implementation of telemedicine [13]. Out of the total number of patients, 230 individuals, which accounts for 85.0% of the sample, expressed satisfaction with their telephone consultation. Patient’s age, sex, and distance to the hospital were not associated with their satisfaction. However, the majority would prefer video consultations in the future if they had a choice in this.

Another crucial factor in assessing the utility of telemedicine is evaluating its immediate impact on monitoring and treating patients to improve the clinical decision-making process. Telemedicine for ureteric colic patients could eliminate the need for face-to-face review consultations, shorten appointment wait times, and better allocate clinic resources to other patients, according to Ong et al. [14]. The percentage of unplanned re-attendance was 3.2% with a sample size of 32. The reason for this was the repeated occurrence of painful colic in 27 cases, as well as miscommunication during the scheduling of the teleconsultation in 5 cases. The positive results of this telemedicine service have been consistently maintained over the past three years since its installation. The monthly recruitment rates ranged from 33.3% to 80.9%. The percentage of clinic consultations saved per month remained steady, ranging from 52.9% to 89.5%. The average time taken to evaluate phone consultations each month ranged from 26.5 to 35.8 days. An innovative telemedicine service for patients with ureteric colic has effectively decreased the frequency of follow-up appointments by 71.1% over three years, greatly exceeding the initial target of 25%. Furthermore, these results demonstrated the sustainability of the service, as seen by a small decline in the participation rate of 4.8%, indicating that patients found this service to be acceptable. This solution optimized resource allocation by saving an average of 238 clinic sessions each year, resulting in reduced waiting times for patients in need of conventional in-person consultations. Cullen et al. define the benefits of a virtual colic clinic for renal colic patients, such as simplified care and shorter wait times [15]. To assess the intervention's efficacy, the duration from referral to clinic review was compared to the proportion of patients seen within four weeks before and after the virtual clinic's deployment. The percentage of non-attendance fell from 18 to 5%. The average time between emergency department referral and urology clinic assessment was reduced from 7.5 to 3.5 weeks. 82% of patients were seen at the clinic within four weeks, up from 25% before. The typical period between referral and intervention ranged from 15 to 5 weeks, including SWL and primary ureteroscopy (URS).

One of the earliest studies examining the application of telemedicine dogma to both the entire urology and endourology fields was described by Johnston et al. who discovered a straightforward way to wirelessly transmit digital CT scans from patients with probable renal colic and one with renal trauma utilizing a Sony Clie 615C hand-held computer and a cellular phone with a modem [16]. Upper-tract stone presence/absence, stone position, estimated stone size, and obstruction indicators were diagnosed. Each patient had 5.9 ± 1.6 pictures, averaging 32.2 ± 5.2 kb (range 21–42 kb), delivered at 1 kb/sec. Interpretation properly recognized 80% of stone presence, 100% hydronephrosis, 80% perinephric stranding, and 1 ± 1 mm stone size. Connor et al. assessed a clinician-led virtual clinic (VC) treatment choice in patients with ureteric colic, with clinical, budgetary, and environmental effects [17]. Clinicians referred patients in real-time utilizing an electronic referral technique integrated into an electronic healthcare records platform (Cerner, North Kansas City, MO, USA). A professional nurse or consultant urologist called the patient on their phone or landline to do a VC consultation. This reduced the median VC decision time to 2 [1–5] days. In total, 347 patients (34.4%) were discharged from the VC (n = 164) or after another VC visit (n = 183). Four patients (0.40%) returned to the emergency room (ER) after a VC due to pain. Direct VC costs were £29,232 and face-to-face (FTF) clinic opportunity costs were £174,384. This saved £145,152 and an estimated 15,085 patient journey kilometers were also averted. Depending on the method of transport, travel avoidance produced 0.70–2.93 metric tons of CO_2_. Hughes et al. detailed their six-year experience with a nurse-led telephone-based virtual stone clinic (VSC) follow-up [18]. This approach was used to monitor patients with asymptomatic renal calculi or those at a high risk of recurring kidney stone disease (KSD). Before the consultation, the patient underwent interval imaging, which included a kidney, ureter, and bladder radiograph (KUB) XR to detect radio-opaque stones, as well as an ultrasound scan (USS) of the urinary tract to identify radiolucent stones. These tests were scheduled at the most convenient time and location for the patient. The VSC was conducted via telephone consultations facilitated by urology-specialized nurses. The duration of telephone consultations normally ranged from 5 to 10 min each. Patients were scheduled for face-to-face consultations if they experienced new symptoms, exhibited stone growth, or expressed a preference for in-person consultations. Over 6 years, the VSC has registered a total of 290 patients, who have collectively attended 468 appointments. By the conclusion of the research period, 132 patients, accounting for 45.6% of the total, were still enrolled in the Virtual Stone Clinic (VSC) and had scheduled follow-up appointments. 106 (36.6%) were discharged, and 47 (16.2%) either returned to in-person clinic visits or required additional treatment. Lastly, 5 (17.%) were hospitalized as emergency cases during the interim period.

To evaluate the effect of telemedicine among patients with an indication for the percutaneous nephrolithotomy (PCNL) treatment, Aydogdu et al. examined how additional video calls affected postoperative results, patient, and surgeon satisfaction in the patients who underwent PCNL [19]. The authors utilized Skype for videoconferencing. The average preoperative telerounding visit lasted 3.65 ± 0.59 (2–4) minutes. Telerounding visits on the first and second postoperative days averaged 3.80 ± 0.62 (2–5) and 2.9 ± 0.91 min. Surgeon satisfaction with telerounding was assessed with a visual analog scale (VAS) score of 91 ± 11.2 (60–100). Patients were satisfied, with 72.5% saying telerounding improved their treatment and 78% saying it should be a regular feature of hospital care. Additionally, 86% of patients said they could easily communicate with their doctor over telerounding, 85.5% said they would feel comfortable telerounding daily if they were hospitalized again, and 79.5% said they would rather use telerounding than see another doctor.

One key benefit of telemedicine is the capacity to monitor patients remotely, potentially resulting in enhanced adherence to physician instructions. Nevo et al. conducted a comparative analysis of the impact of telephone communication (PC) and in-person office consultations (OC) on urinary metabolic risk factors associated with stone formation [20]. The overall number of visits and the duration between the initial consultation and the final visit varied considerably between the groups. In comparison to patients who only underwent OC visits, those who underwent PC and OC had extended follow-up periods (51.7 vs 18.5 months, p < 0.0001) and more consultations (OC or PC, 5.4 vs 2.5, p < 0.0001). 86% of patients with OC alone had 1:3 return consultations, whereas 90% of patients with either OC or PC had 2:8 return consultations. A stone recurrence was observed in eight patients (38%) who had both OC and PC, compared to six (27%) patients who had only OC (p = 0.52). Notably, patients in the telemedicine group had substantially greater increases in urine volume than those in the OC group.

A study by Sarmah et al. dedicated specifically to the financial benefits obtained from performing telephone follow-up with patients following BOO surgery [21]. With VC, the total cost for the fifty telephone sessions was £2,392. Eleven patients who were not discharged promptly incurred supplementary fees totaling £3,674 for uroflowmetry and FTF visits. £6,066 was the entire cost of the new service. The projected cumulative cost savings to the health sector over a ten-month duration resulting from the utilization of virtual consultations amounted to £10,634. Three months after their surgery, patients were allocated a specific date and time for their appointments, and subsequently, they were contacted via telephone, contingent upon the realization of a post-operative trial without a catheter (TWOC). The patients were contacted via telephone to complete the IPSS questionnaire; the IPSS and Quality of Life (QoL) scores were calculated based on their responses. Primary care was notified of patients whose IPSS scores fell below eight.

## Discussion

### Pros of Telemedicine in Endourology

This systematic review examines the impact of telemedicine in endourology. Each study identified a multitude of benefits associated with this technology. This supports the notion that telemedicine is swiftly integrating itself into the healthcare sector and gaining favor among physicians and patients. Undoubtedly, the examination of the studies incorporated in the review is adequate to ascertain the advantages of telemedicine throughout the diagnostic and therapeutic processes for patients and endourologists. Thus, telemedicine is a feasible alternative for initial or subsequent visits, and it is especially beneficial for patients who are hindered by geography or socioeconomic status. This enables ureteric colic patients to have shorter review periods and in-person consultations while maintaining the highest standards of safety.

An assessment of the efficacy of telemedicine can reveal two significant benefits: an increase in the availability of space in clinics and parking areas, and a reduction in greenhouse gas emissions [9, 11, 14]. Furthermore, telemedicine decreases waiting periods for treatment and clinic reviews, expands the capacity of clinics, and reduces the financial burden on clinics [15–18]. Finally, endourology patients represent a favorable cohort for the adjunctive utilization of telemedicine in the context of their diagnosis and treatment. Despite the availability of in-person session scheduling options, patients who underwent telemedicine consultations for kidney stones and pain reported significant levels of satisfaction with their telemedicine appointments. Including telerounding in endourological patient care improves surgeon and patient satisfaction, as well as subsequent appointment adherence [12, 13, 19, 20].

### Limitations of Telemedicine in Endourology

On the contrary to the advantages, very few articles mentioned the drawbacks of telemedicine as they pertained to endourology. This dearth of literature hinders a comprehensive evaluation of the drawbacks and constraints, necessitating further literature review. The examination of the chosen studies underscores the fact that the effective implementation of telemedicine, which was previously regarded as essential in the education of healthcare professionals and clinicians, is contingent upon the competence of a highly trained physician in patient assessment and, at times, the use of specialized equipment [10, 13, 16, 18]. This may result in inequitable care for those without access, thereby exacerbating healthcare disparities. The use of telemedicine necessitates reliable internet connections and appropriate devices for medical practitioners and patients alike. As was shown by Checcucci et al. 53% (322/607) of interviewees lacked the basic equipment needed for telemedicine consultations. 68% (413/607) said they would not have been able to complete an online visit to the needed standards even if they had the chance [22].

Moreover, in contrast to the preceding assertion concerning the overall acceptance of telemedicine among endourology patients, certain subgroups within the field are more receptive to this technology. This underscores the significance of examining the methods by which endourology patients comply with telemedicine. Individuals with urologic conditions associated with infertility were found to utilize telemedicine at a higher rate than those with general urology/endourology, female urology, urologic oncology, or reconstructive surgery, according to a study by Javier-DesLoges et al. This is owing in part to the demographic characteristics of endourological patients in contrast to andrological patients. As an illustration, the former group exhibits a higher propensity for advanced age, substantial comorbidities, and intricate pathology [11, 17]. In the digital age, protecting patient data is crucial to maintaining patient trust and complying with strict standards like ‘Health Insurance Portability and Accountability Act’ (HIPAA) in the US. The EU also enforces strict data privacy and electronic record requirements through the General Data Privacy Regulation (GDPR). In the age of interconnected health systems, telemedicine requires the tightest data privacy and security.

Telemedicine's complex and changing regulatory environment should be considered. The American Urological Association (AUA) recommends that urologists continue using telemedicine since it will continue to be important and durable in healthcare delivery and urology education [23]. To ensure that the patient has the necessary records and can attend their appointment, clinic personnel should send text messages and email reminders. As with an in-person session, the clinical record must include all relevant information to meet the highest clinical standards. The letter should specify, "Teleconsultation is offered exclusively upon the patient's consent" [24]. Kirshenbaum et al. studied the Centers for Medicare & Medicaid Services (CMS)'s latest telemedicine regulatory overhaul [25]. The 2021 regulations prioritize medical decision-making (MDM) in service-level assessments. Certain medical history and physical exam components are no longer required. The service-level decision relies solely on MDM by providers.

## Conclusion

Telemedicine is a promising tool in endourology that has shown its usefulness not only during the pandemic but also in the subsequent period. It offers the potential to reduce patient travel time, expedite decision-making, and save costs. Implementing telemedicine in everyday practice is challenging due to various obstacles faced by patients and providers, hindering the realization of its full potential and necessitating a systematic approach to problem-solving.

## Data Availability

No datasets were generated or analysed during the current study.
